# SP600125 Enhances Temperature-Controlled Repeated Thermal Stimulation-Induced Neurite Outgrowth in PC12-P1F1 Cells

**DOI:** 10.3390/ijms232415602

**Published:** 2022-12-09

**Authors:** You-Ran Luo, Tada-aki Kudo, Kanako Tominami, Satoshi Izumi, Takakuni Tanaka, Yohei Hayashi, Takuya Noguchi, Atsushi Matsuzawa, Junichi Nakai, Guang Hong, Hang Wang

**Affiliations:** 1 State Key Laboratory of Oral Diseases, National Clinical Research Center for Oral Diseases, West China Hospital of Stomatology, Sichuan University, Chengdu 610041, China; 2 Division for Globalization Initiative, Tohoku University Graduate School of Dentistry, Sendai 980-8575, Japan; 3 Division of Oral Physiology, Tohoku University Graduate School of Dentistry, Sendai 980-8575, Japan; 4 Cell Resource Center for Biomedical Research, Institute of Development, Aging and Cancer, Tohoku University, Sendai 980-8575, Japan; 5 Graduate School of Life Sciences, Tohoku University, Sendai 980-8577, Japan; 6 Laboratory of Health Chemistry, Graduate School of Pharmaceutical Sciences, Tohoku University, Sendai 980-8578, Japan

**Keywords:** PC12 cells, temperature-controlled repeated thermal stimulation, neuritogenesis, SP600125

## Abstract

This study evaluated the mechanism of temperature-controlled repeated thermal stimulation (TRTS)-mediated neuronal differentiation. We assessed the effect of SP600125, a c-Jun N-terminal kinase (JNK) inhibitor, on neuronal differentiation of rat PC12-P1F1 cells, which can differentiate into neuron-like cells by exposure to TRTS or neurotrophic factors, including bone morphogenetic protein (BMP) 4. We evaluated neuritogenesis by incubating the cells under conditions of TRTS and/or SP600125. Cotreatment with SP600125 significantly enhanced TRTS-mediated neuritogenesis, whereas that with other selective mitogen-activated protein kinase (MAPK) inhibitors did not—e.g., extracellular signal-regulated kinase (ERK)1/2 inhibitor U0126, and p38 MAPK inhibitor SB203580. We tried to clarify the mechanism of SP600125 action by testing the effect of U0126 and the BMP receptor inhibitor LDN193189 on the SP600125-mediated enhancement of intracellular signaling. SP600125-enhanced TRTS-induced neuritogenesis was significantly inhibited by U0126 or LDN193189. Gene expression analysis revealed that TRTS significantly increased β3-Tubulin, MKK3, and Smad7 gene expressions. Additionally, Smad6 and Smad7 gene expressions were substantially attenuated through SP600125 co-treatment during TRTS. Therefore, SP600125 may partly enhance TRTS-induced neuritogenesis by attenuating the negative feedback loop of BMP signaling. Further investigation of the mechanisms underlying the effect of SP600125 during TRTS-mediated neuritogenesis may contribute to the future development of regenerative neuromedicine.

## 1. Introduction

Neurite outgrowth is a key process in nervous system regeneration [1]. Thus, understanding extracellular signals capable of inducing or enhancing neuritogenesis is crucial for developing therapeutic treatments for neurological disorders [2,3,4]. The PC12 rat pheochromocytoma cell line is most commonly used as a model for studying neuronal differentiation and its signaling pathways [5,6]. PC12 cells respond to a variety of neurotrophic factors, such as nerve growth factor (NGF) and bone morphogenetic proteins (BMPs), and they differentiate into sympathetic neuron-like phenotypes characterized by neurite outgrowth and the expression of many neuronal-specific proteins [7,8].

Mitogen-activated protein kinases (MAPKs) are a group of serine–threonine kinases that include extracellular signal-regulated kinases (ERK1, ERK2, and ERK5), c-Jun N-terminal kinases (JNK1, JNK2, and JNK3), and p38 MAPKs (p38α, p38β, p38γ, and p38δ) [9]. The four p38 MAPK isoforms are encoded by different genes and have different tissue expression patterns, with p38α being ubiquitously expressed at significant levels in most cell types, whereas the other isoforms appear to be expressed in a more tissue-specific manner; for example, p38β in the brain, p38γ in the skeletal muscle, and p38δ in the endocrine glands [10,11]. In general, these MAPK family members are phosphorylated and activated by upstream kinases, i.e., MAPK kinases (MAP2Ks). For example, in the classical pathway, p38 is activated by critical MAP2Ks, MKK3, and MKK6 (MKK3/6) [12]. However, MKK3 can reportedly activate p38α, p38γ, and p38δ, but not p38β, whereas MKK6 can activate four p38 MAPK isoforms [11]. Furthermore, it is interesting to note that p38 can also be activated through the other atypical pathways, independent from MKK3/6 [13].

MAPKs mediate intracellular signaling associated with various critical cellular activities—i.e., cell proliferation, differentiation, transformation, survival, and death [14,15]. Furthermore, NGF signaling mediated by tropomyosin receptor kinase A induces neurite outgrowth by rapidly and sustainably activating ERK1/2 [16]. Besides ERK1/2, ERK5 is essential for neurite outgrowth [17] and may contribute to neuronal differentiation by phosphorylating a voltage-gated potassium channel, i.e., Kv4.2 in PC12 cells [18]. Furthermore, p38 MAPK is required for neuritogenesis induced by ligands, such as BMPs, in PC12 cells [19].

Although JNK-mediated phosphorylation of the transcription factor c-Jun is critical for cell survival and death [20], the role of JNK in neuronal differentiation in PC12 cells remains largely unclear. The study results using PC12-N1, a subclone of the PC12 cell line that spontaneously elongates neurites without the addition of neurotrophins [21], have implied that JNK activation might play a role in NGF-induced neurite-like process elongation [22].

BMPs belong to the transforming growth factor-β (TGF-β) cytokine superfamily, which mediates various biological events, including neuronal development [23]. For example, BMP2 and BMP4 cause neurite outgrowth in PC12 cells in a different method from that induced by NGF [4,24]. Moreover, BMP4 induces neuritogenesis in E9 chicken embryos [25] and increases NGF-mediated neurite outgrowth in PC12 cells [26]. BMP receptors are classified into two types of transmembrane receptors as follows: type I and type II. After a complex formation among BMPs, types I and II BMP receptors, type II receptors phosphorylate and activate type I receptors, which consequently activates the following two downstream pathways: the Smad and the TGF-β-associated kinase 1 (TAK1)-p38 MAPK (non-Smad) signaling pathways, respectively [27]. In the case of the BMP-induced neuronal differentiation of PC12 cells, neurite outgrowth is at least dependent on BMP-mediated TAK1-p38 MAPK signaling [19,28,29]. In this context, TAK1, a member of the MAP2K kinase (MAP3K) family, was originally identified as a protein kinase activated by TGF-β and BMPs [30].

In the Smad signaling pathway, the receptor-regulated Smad (R-Smad) proteins Smad1, Smad5, and Smad8, which are phosphorylated and activated by type I receptors, translocate to the nucleus and are associated with the common-partner Smad (Co-Smad) protein, Smad4 [28]. The R-Smad and Co-Smad complex then act as transcription factors, resulting in the transactivation of target genes, including the inhibitory Smads (I-Smads), Smad6, and Smad7 [31]. Owing to the fact that I-Smads can interact with activated BMP type I receptors and thereby prevent the activation of R-Smads or I-Smads can compete with activated R-Smads for heteromeric complex formation with Smad4, I-Smads can work as inhibitors to inactivate the Smad pathway and BMP receptor-mediated TAK1-p38 MAPK pathway [27,28].

Notably, the above-mentioned neurotrophins (NGF and BMPs) have limitations in terms of humoral factors, namely their rapid diffusion rate and short half-life in vivo [32]. Therefore, a novel method using thermal stimulation may be a potential alternative to neurotrophin treatment and useful for neuronal regeneration. The heat shock stimulation has been reported to significantly affect in vitro neurite outgrowth in PC12m3 cells, a subclone of the PC12 cell line [33]. Furthermore, short-term heat shock stimulation of PC12m3 cells enhanced NGF-mediated neuronal differentiation and only required p38 MAPK, not ERK1/2, signaling [34].

Additionally, our previous studies reported that temperature-controlled repeated thermal stimulation (TRTS), which is a programmed sequence of mild thermal stimulation, significantly induces in vitro neuritogenesis of PC12 cells without the addition of NGF or BMPs [4]. We previously established two new subclones from PC12 parental cells according to their sensitivity to TRTS in an effort to efficiently clarify the mechanism of TRTS-mediated neuronal differentiation at the cellular and molecular level [35]. One of the PC12 subclones (PC12-P1F1) had a higher potential for neuritogenesis under TRTS compared with the PC12 parental cell line, while the other subclone (PC12-P1D10) was unable to elongate neurites upon TRTS treatment.

In this current study, we pharmacologically investigated the role of various MAPK signaling pathways in TRTS-induced neuritogenesis of the PC12-P1F1 subclone by pretreating the cells with a number of MAPK inhibitors. We report the unexpected finding that SP600125, a selective inhibitor of JNK, enhances the neurite outgrowth of PC12-P1F1 cells. This property may be useful for developing a more efficient method of TRTS in the future.

## 2. Results

### 2.1. Regulation of TRTS

**Figure 1:**
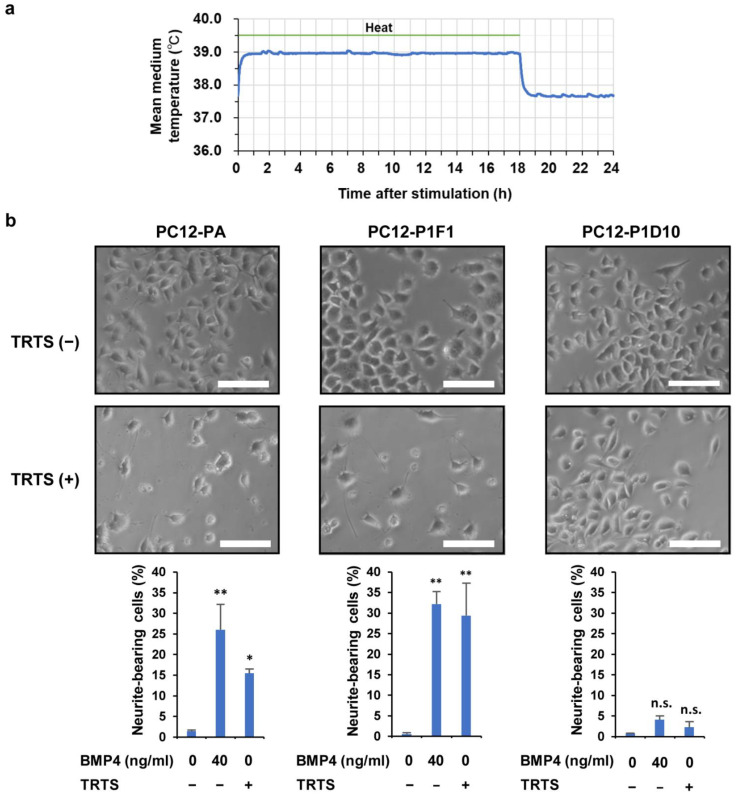
Comparison of temperature-controlled repeated thermal stimulation (TRTS)-induced neurite-bearing cells (%) among PC12 parental, PC12-P1F1, and PC12-P1D10 cell lines. (**a**) We evaluated temperature changes in the culture medium during TRTS. Briefly, 24 h before thermal evaluation, a cell-free culture medium was transferred into a 24-well culture plate. Then, temperatures of the culture medium during TRTS (18 h/day) were recorded every 60 s for 24 h. The data represent the average temperature changes of four independent replicates. (**b**) Cells were exposed to TRTS for 18 h per day for 7 days, and the percentage of neuritogenesis was evaluated. Representative phase-contrast micrographs of cultured cells and the data of neurite-bearing cells on day 7 with or without TRTS of three cell lines: PC12 parental cells (PC12-PA), PC12-P1F1 cells, and PC12-P1D10 cells. Scale bars: 100 μm. The data represent the means ± standard deviation of three replicates. * *p* < 0.05 vs. control; ** *p* < 0.01 vs. control; n.s., not significant vs. control.

The temperature of the culture medium in the well of a 24-well culture plate was measured in the presence and absence of TRTS. TRTS was generated by a heating plate that was placed under the culture plate. We confirmed the effect of TRTS on the temperature of the medium and also evaluated the thermal stability of TRTS. Upon initiation of the TRTS exposure programmed to constantly stimulate cells for 18 h per day, the culture medium temperature rapidly increased to 39 °C and was maintained for 18 h during heating. When the heating plate automatically switched off after 18 h of heating, the temperature of the medium decreased to an equilibrium temperature of 37.7 °C (Figure 1a), similar to our previous study [4,35]. These results indicate that the culture medium temperature was mechanically regulated in a rigorously reproducible manner utilizing the TRTS program.

To confirm the responses of the three cell lines (PC12 parental cells, PC12-P1F1 cells, and PC12-P1D10 cells) to the TRTS program (Figure 1a), each cell line was exposed to TRTS for 7 days before evaluating the percentage of neurite-bearing cells. Before TRTS exposure (day 0), the cells were relatively round and small, with few visible neurites. In contrast, on day 7, TRTS-mediated neurite outgrowth was observed in PC12 parental cells and PC12-P1F1 cells, even in the absence of neurotrophic chemical inducers. As expected, a more significant increase in TRTS-induced neuritogenesis occurred in PC12-P1F1 cells compared with PC12 parental cells, while no significant neuritogenesis was observed in PC12-P1D10 cells on day 7, despite TRTS exposure (Figure 1b). Thus, we selected the PC12-P1F1 subclone as the main experimental model for subsequent experiments in this study, mainly due to its stronger reaction to TRTS treatment, which might contribute to the efficient analysis of TRTS-mediated neuritogenesis and neuronal differentiation.

### 2.2. Promotion of TRTS-Mediated Neuritogenesis by SP600125 Treatment

**Figure 2:**
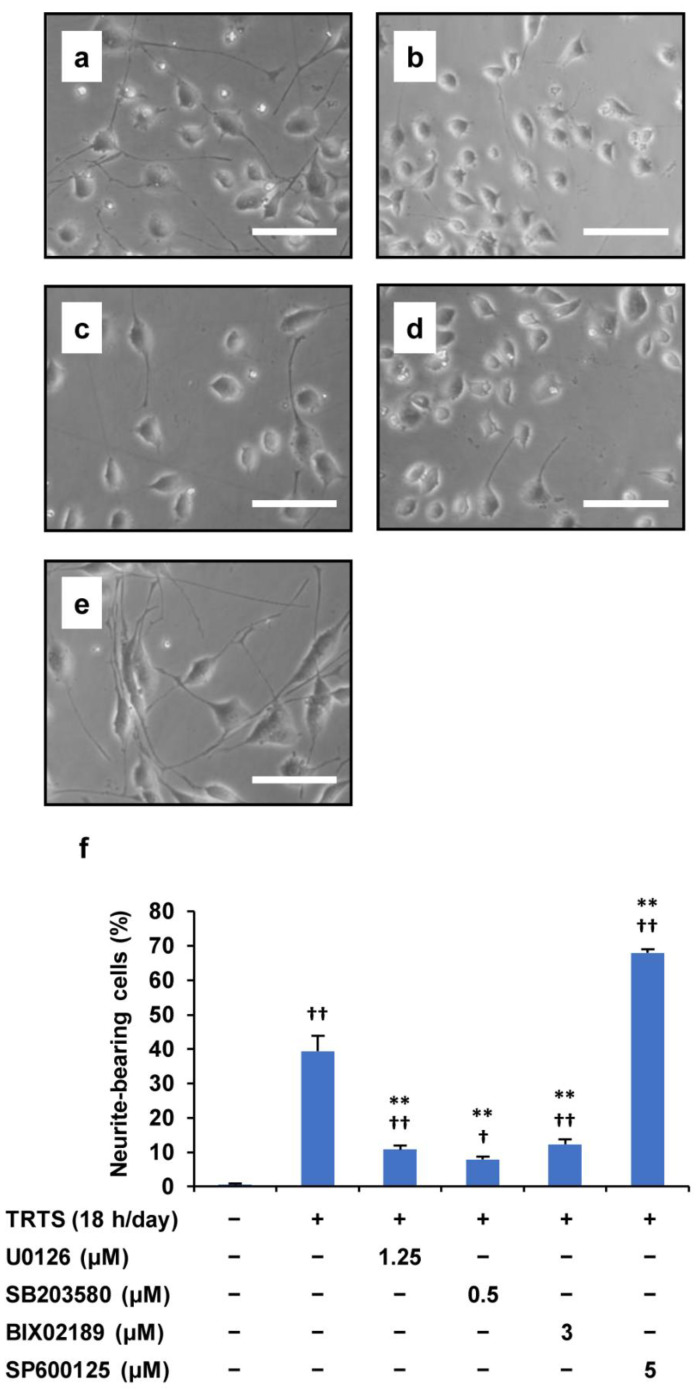
Effect of MAPK inhibitors on TRTS-induced differentiation in PC12-P1F1 cells. PC12-P1F1 cells were pretreated with MAPK inhibitors before TRTS. (**a**–**e**) Phase-contrast images of the cells on day 7 after TRTS alone (**a**) or pretreated with U0126 (**b**), BIX02189 (**c**), SB2003580 (**d**), or SP600125 (**e**). Scale bars: 100 μm. (**f**) PC12-P1F1 cells were exposed to TRTS for 18 h per day for 7 days with or without pretreatment with MAPK inhibitors, and a control group was incubated with no inhibitor and no TRTS exposure. The percentage of neurite-bearing cells on day 7 was determined. The data represent the means ± standard deviation of three replicates. ^†^ *p* < 0.05 vs. control; ^††^ *p* < 0.01 vs. control; ** *p* < 0.01 vs. TRTS alone.

In our previous study, we already showed that the ERK1/2 and p38 MAPK signaling pathways might participate in TRTS-mediated neuronal differentiation in PC12 parental cells [4], prompting us to investigate the role of various MAPKs in TRTS-induced differentiation of PC12-P1F1 subclones, previously developed by us for a more efficient TRTS research. With this background, to further investigate the mechanism of the TRTS-induced neuronal differentiation in the present study, we treated PC12-P1F1 cells with four types of inhibitors during TRTS-mediated neuronal differentiation: (i) U0126, an ERK1/2 kinase inhibitor that blocks the upstream MAPK/ERK kinase (MEK) 1/2; (ii) SB2003580, which inhibits p38 MAPK; (iii) BIX02189, which inhibits ERK5 activity by blocking the ERK5 kinase MEK5, and (iv) SP600125, which inhibits JNK. Treatment with U0126, SB2003580, or BIX02189 substantially decreased the extent of TRTS-induced neuritogenesis in PC12-P1F1 cells. In contrast, and most unexpectedly, the JNK inhibitor SP600125 considerably increased the percentage of neurite-bearing cells (up to 67.9%) compared with the TRTS-exposed cells in the absence of any types of MAPK inhibitors (up to 39.3%) (Figure 2).

### 2.3. Time Course and Morphological Characteristics of TRTS Plus SP60015-Induced Neuritogenesis

We showed that SP600125 can promote TRTS-mediated neuritogenesis in PC12-P1F1 cells. We consequently evaluated the time course of SP600125-enhanced TRTS-induced neuritogenesis in PC12-P1F1 cells. PC12-P1F1 cells or PC12-P1D10 cells (as a control) were scored for neurite outgrowth at the indicated times (days 0, 3, 5, or 7) after adding TRTS (18 h/day) in the presence or absence of 5.0 μM SP600125 or 40 ng/mL BMP4. BMP4 was used as a possible positive control of TRTS-effect enhancer. To compare the time course of TRTS plus SP600125-mediated neuritogenesis with that of other inducers in the employed PC12 subclones (PC12-P1F1 and PC12-P1D10 cells), the subclones were also incubated with BMP4 alone (40 ng/mL) or NGF alone (50 ng/mL) for 7 days. Furthermore, to evaluate the morphological changes of incubated cells with various stimulations, the live cells were also specifically visualized with fluorescence diacetate (FDA) on day 0 before starting various stimulations or day 7 at post-treatment with various stimulations.

**Figure 3:**
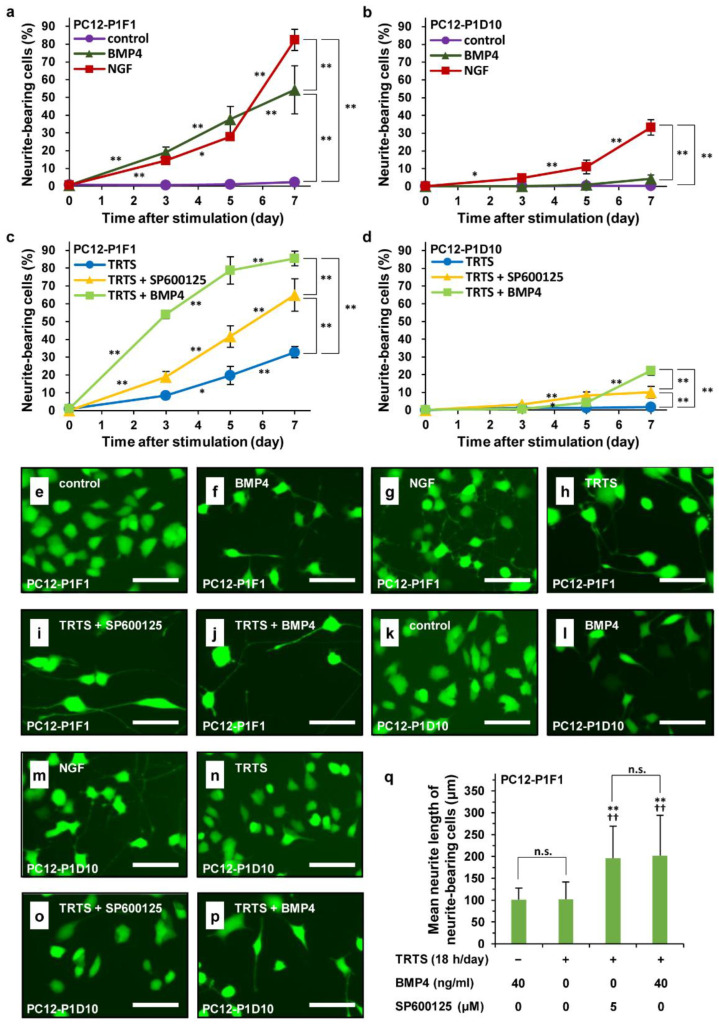
Time course and live imaging of SP600125-mediated enhancement of TRTS-induced neuritogenesis in PC12-P1F1 cells. PC12-P1F1 or PC12-P1D10 cells were incubated with BMP4 (40 ng/mL) or NGF (50 ng/mL) for 7 days. Furthermore, PC12-P1F1 or PC12-P1D10 cells were also pretreated with SP600125 (5.0 μM) or BMP4 (40 ng/mL) with TRTS 18 h/day for 7 days. PC12-P1F1 (**a**,**c**) and PC12-P1D10 cells (**b**,**d**) were scored for neurite outgrowth after 0–7 days of incubation with the indicated conditions. (**a**–**d**) The data represent the means ± standard deviation of four replicates. * *p* < 0.05; ** *p* < 0.01. (**e**–**j**) Representative live images of PC12-P1F1 cells treated without stimuli (a control) (**e**), BMP4 alone, (**f**), NGF alone (**g**), TRTS alone (**h**), TRTS plus SP600125 (**i**), and TRTS plus BMP4 (**j**). (**k**–**p**) Representative live cell images of PC12-P1D10 cells treated without stimuli (a control) (**k**), BMP4 alone, (**l**), NGF alone (**m**), TRTS alone (**n**), TRTS plus SP600125 (**o**), and TRTS plus BMP4 (**p**). (**e**–**p**) Scar bars: 100 μm. (**q**) The averaged neurite length of neurite-bearing PC12-P1F1 cells 7 days after the indicated stimulations. The data represent the means ± standard deviation of ten replicates. ^††^ *p* < 0.01 vs. BMP alone; ** *p* < 0.01 vs. TRTS alone; n.s., not significant.

As shown in Figure 3a–d, TRTS plus SP600125-mediated neurite outgrowth occurred in a time-dependent manner (up to 64.9%) and more rapidly than TRTS-alone-induced neurite outgrowth (up to 32.8%) in PC12-P1F1 cells. However, in PC12-P1D10 cells that almost lose the ability to elongate neurite in response to TRTS or BMP4 (Figure 1b) [35], despite the same experimental conditions, the extent of neuritogenesis was limited and the rate of neurite-bearing cells only reached 10% even in the co-treatment of SP600125 in the course of neuronal differentiation. On the other hand, we found that the co-treatment of BMP4 with TRTS also induced neuritogenesis in a time-dependent manner and significantly enhanced TRTS-induced neuritogenesis in PC12-P1F1 cells (from 32.8% to 85.4%), showing that BMP4 can also upregulate the neuritogenesis induced by TRTS, similar to SP600125. However, such as in the case of TRTS plus SP600125, the percentage of neurite-bearing PC12-P1D10 cells at post-treatment with TRTS plus BMP4 for 7 days was much lower (up to 22.2%) than that of PC12-P1F1 (up to 85.4%). Moreover, as shown in Figure 3e–q, the live cell imaging using FDA also showed that live neurite-bearing PC12-P1F1 cells treated with SP600125 or BMP4 in addition to TRTS for 7 days had near two times longer neurites compared to neurites of neurite-bearing PC12-P1F1 cells only treated with BMP4 or TRTS alone for 7 days.

### 2.4. Effects of Dose and Treatment Time of SP600125 on TRTS-Induced Neuritogenesis

**Figure 4:**
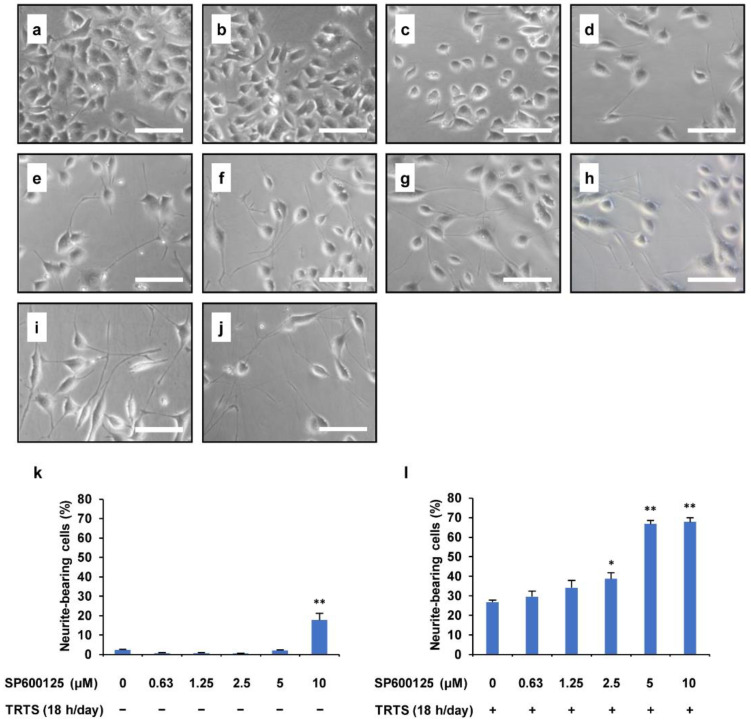
Dose-dependent SP600125-mediated enhancement of TRTS-induced neuritogenesis in PC12-P1F1 cells. PC12-P1F1 cells were pretreated with SP600125 (0–10 μM) with or without TRTS (18 h/day for 7 days). (**a**–**d**) Representative phase-contrast images of PC12-P1F1 cells treated with the indicated concentrations of SP600125 in the absence of TRTS: 0 μM (**a**), 2.5 μM (**b**), 5 μM (**c**), and 10 μM (**d**). (**e**–**j**) Representative phase-contrast images of PC12-P1F1 cells on day 7 incubated with the indicated concentrations of SP600125 in the presence of TRTS: 0 μM (**e**), 0.625 μM (**f**), 1.25 μM (**g**), 2.5 μM (**h**), 5 μM (**i**), and 10 μM (**j**). Scale bars: 100 μm. (**k**,**l**) PC12-P1F1 cells were scored for neurite outgrowth after 7 days of incubation. The data represent the means ± standard deviation of three replicates. * *p* < 0.05 vs. control; ** *p* < 0.01 vs. control (**k**), or TRTS alone (**l**).

The observed enhancement of TRTS-induced neuritogenesis in PC12-P1F1 cells by SP600125 prompted us to further evaluate the dose–response effect of SP600125 in the current PC12-P1F1 subclone differentiation model. Therefore, we determined the percentage of neurite-bearing PC12-P1F1 cells with or without TRTS exposure for 7 days with increasing concentrations of SP600125 (0–10 μM). As shown in Figure 4, when PC12-P1F1 cells were treated with SP600125 alone and at a concentration of ≤5 μM, there was no statistically significant induction of neuritogenesis. However, treatment with 10 μM SP600125 alone induced significant neurite outgrowth on approximately 17% of neurite-bearing cells. During TRTS-induced neuronal differentiation of PC12-P1F1 cells for 7 days, SP600125 treatment significantly enhanced the degree of neuritogenesis in a dose-dependent manner. Treatment with 5 μM SP600125 resulted in an obvious increase in TRTS-induced neurite outgrowth on approximately 70% of neurite-bearing cells. However, treatment with 10 μM SP600125 did not significantly increase the rate of differentiation compared with treatment with 5 μM SP600125. Thus, we decided to use 5 μM as the standard concentration for SP600125 treatment in the subsequent investigations in this study.

**Figure 5:**
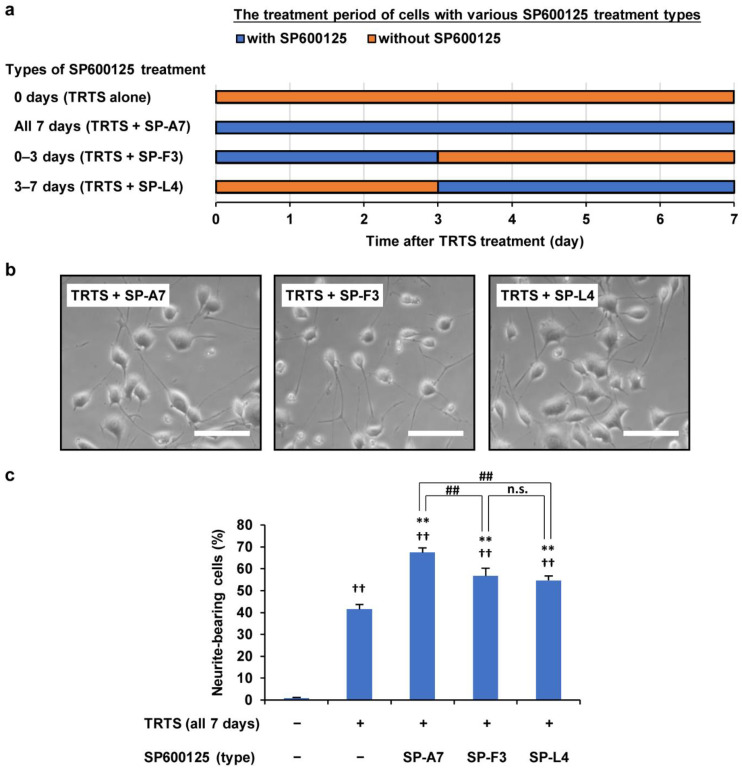
Effects of various treatment times with 0.5 μM SP600125 on PC12-P1F1 neuritogenesis while undergoing TRTS exposure. (**a**) Schematic representation of the treatment times of PC12-P1F1 cells with SP600125 in the presence of TRTS. PC12-P1F1 cells were stimulated with SP600125 under TRTS exposure as follows: all 7 days (TRTS + SP-A7), first 3 days (TRTS + SP-F3), and last 4 days (TRTS + SP-L4). (**b**) Phase-contrast images of PC12-P1F1 cells on day 7. Scale bars: 100 μm. (**c**) Percentage of neurite-bearing cells on day 7. Cells that did not undergo TRTS or SP600125 treatment were defined as the negative control group. The data represent the means ± standard deviation of three replicates. ^##^ *p* < 0.01; n.s., not significant; ^††^ *p* < 0.01 vs. control; ** *p* < 0.01 vs. TRTS alone.

Next, we assessed the possibility that varying the SP600125 treatment time might change the extent of the TRTS-induced neuritogenesis in PC12-P1F1 cells. For this purpose, we scored the ratio of neurite-bearing cells on day 7 in PC12-P1F1 cells treated with TRTS in the presence of 5 μM SP600125 for the following periods: (i) 0 days (TRTS alone), (ii) all 7 days (TRTS + SP-A7), (iii) first 3 days (TRTS + SP-F3), and (iv) last 4 days (TRTS + SP-L4, i.e., SP600125 was added on day 3 of TRTS-induced neuronal differentiation) (Figure 5a). Additionally, as a negative control, cells were also incubated with neither SP600125 nor TRTS for 7 days. The various SP600125 treatment conditions showed significant promotion of TRTS-induced neuritogenesis in all cases except for 0 days (TRTS alone). However, the partial treatment with SP600125 during TRTS exposure (TRTS + SP-F3 and TRTS + SP-L4) significantly decreased neurite outgrowth compared with TRTS + SP-A7, implying that the constant presence of SP600125 during TRTS-treated neuronal differentiation is essential for achieving more efficient TRTS-dependent neuritogenesis (Figure 5b,c).

### 2.5. Relationship between the SP600125-Mediated Promoting Effect and the Target of SP600125

**Figure 6:**
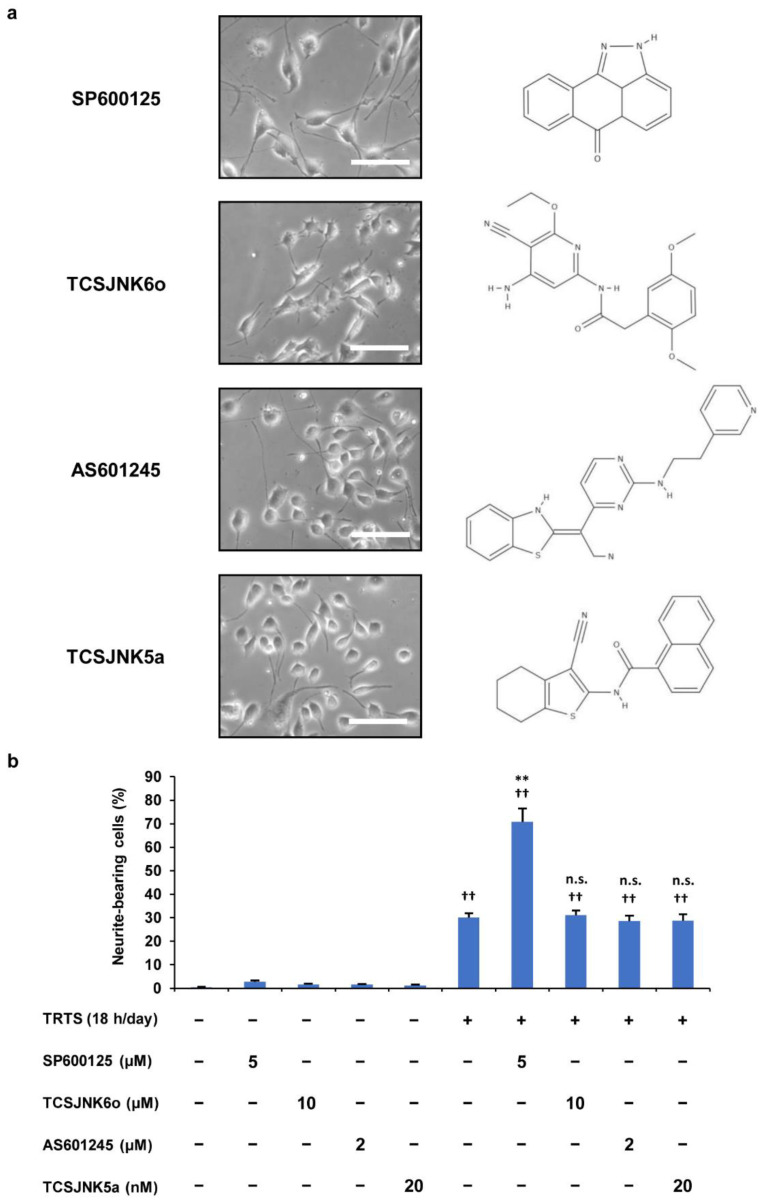
Effects of SP600125 and the other JNK inhibitors (TCSJNK6o, AS601245, and TCSJNK5a) on TRTS-mediated neuritogenesis in PC12-P1F1 cells. PC12-P1F1 cells were pretreated with 5.0 μM SP600125, 10 μM TCSJNK6o, 2.0 μM AS601245, or 20 nM TCSJNK5a and then exposed to TRTS for 18 h per day for 7 days. (**a**) Representative phase-contrast images of PC12-P1F1 cells on day 7 in the presence of TRTS and the chemical structures of the indicated JNK inhibitors. Scale bars: 100 μM. (**b**) The percentage of neurite-bearing cells on day 7 was counted using microscopy. The data represent the means ± standard deviation of three replicates. ^††^ *p* < 0.01 vs. control; ** *p* < 0.01 vs. TRTS alone; n.s., not significant vs. TRTS alone.

To further elucidate the role of SP600125 that was found to be involved in the neuronal differentiation of PC12-P1F1 cells, we next attempted to clarify whether other JNK inhibitors showed the same promoting effects. For this experiment, the TRTS-induced differentiation of PC12-P1F1 cells was performed after treatment with SP600125 or one of the following JNK inhibitors: TCSJNK6o, AS601245, or TCSJNK5a. We scored PC12-P1F1 cells for neurite outgrowth for 7 days after TRTS stimulation with or without the above JNK inhibitors. In contrast to SP600125, other inhibitors (TCSJNK6o, AS601245, and TCSJNK5a) failed to significantly enhance the TRTS-induced neurite outgrowth of PC12-P1F1 cells (Figure 6a,b). These observations led us to consider the possibility that inhibition of the JNK signaling pathway followed by the selective inhibition of JNK activity by SP600125 might not be the primary reason for the observed SP600125-mediated promotion of TRTS-induced neurite elongation in PC12-P1F1 cells. Furthermore, the above results also raised the possibility that SP600125 might have another unknown target molecule(s) in PC12-P1F1 cells that mediate the observed promoting effect of the novel neuritogenesis enhancer SP600125 that we first reported in this study.

**Figure 7:**
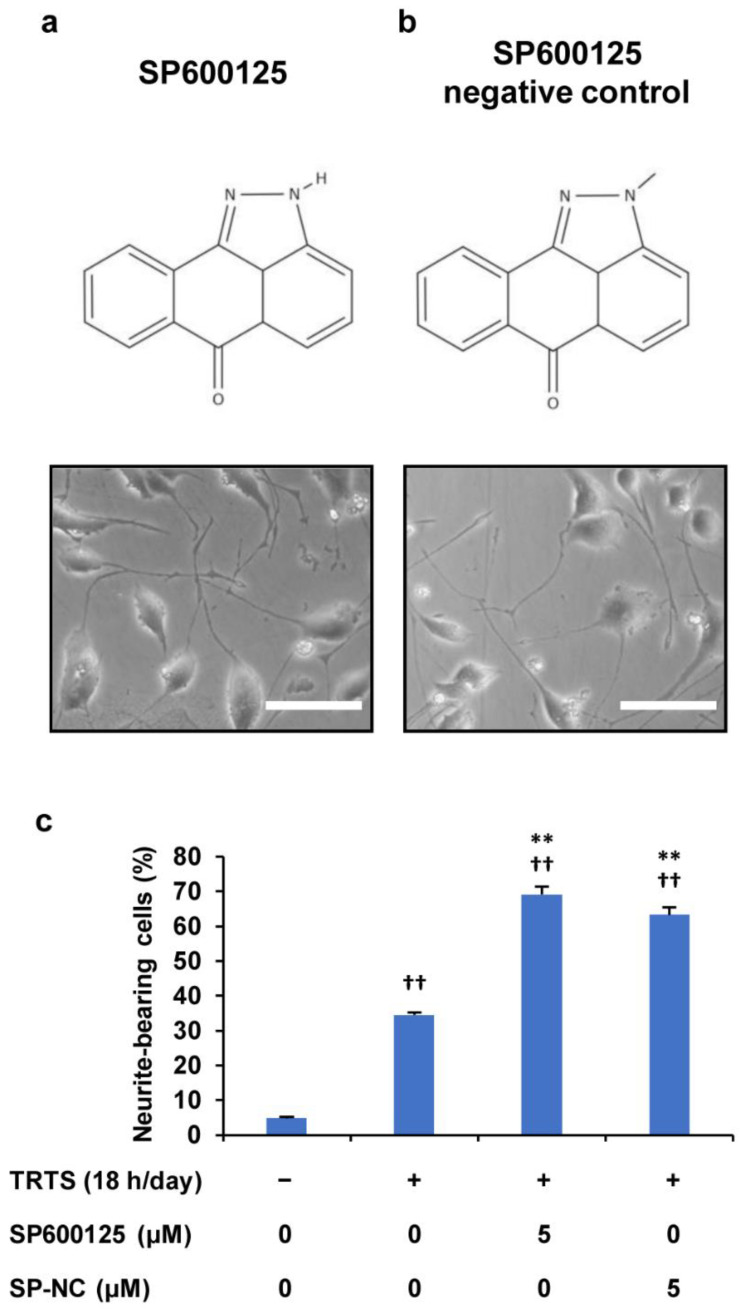
Effect of SP600125 negative control (SP-NC) on TRTS-mediated neuritogenesis in PC12-P1F1 cells. PC12-P1F1 cells were pretreated with 0.5 μM SP600125 and SP-NC, respectively, and then exposed to TRTS for 18 h per day for 7 days. (**a**,**b**) Chemical structures of SP600125 (**a**) and SP-NC (**b**) and representative phase-contrast images on day 7 of TRTS-treated PC12-P1F1 cells in the presence of each compound. Scale bars: 100 μm. (**c**) Percentage of neurite-bearing cells on day 7. The data represent the means ± standard deviation of three replicates. ^††^ *p* < 0.01 vs. control; ** *p* < 0.01 vs. TRTS alone.

In an attempt to elucidate these issues, we next assessed the effect of the chemical compound SP600125 negative control (SP600125-NC), which is an analog of SP600125 that lost the ability to inhibit JNK activity, by comparing it with SP600125. For this experiment, PC12-P1F1 cells were pretreated with 5 μM SP600125 and 5 μM SP600125-NC, respectively, and then the cells were exposed to TRTS (18 h/day) for 7 days. The results showed that compared with SP600125, SP600125-NC had a similar promoting effect on TRTS-induced neuritogenesis in PC12-P1F1 cells (Figure 7), which strongly supports the above-described idea of SP600125 potentially targeting another unknown molecule(s) in the cells.

### 2.6. Suppression of the SP600125-Mediated Promotion of TRTS-Induced Neuritogenesis by U0126 and LDN193189

**Figure 8:**
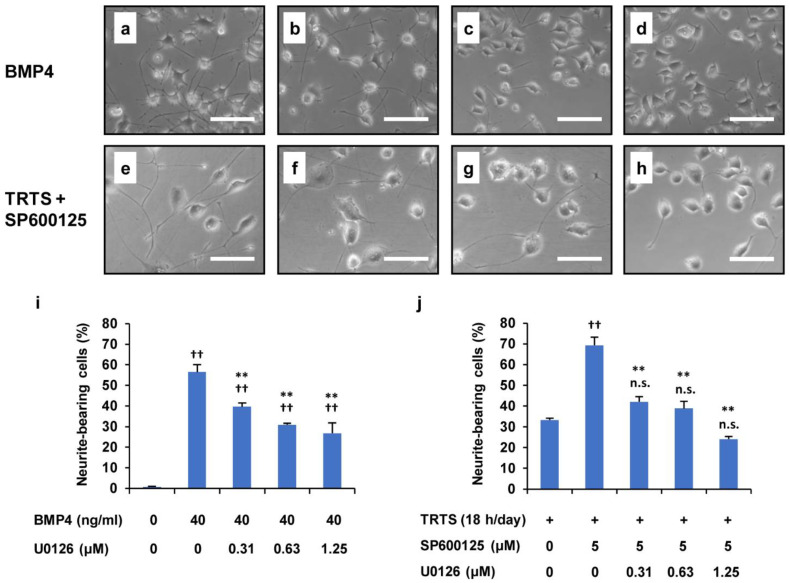
Suppression of TRTS plus SP600125-mediated neuritogenesis by U0126 in PC12-P1F1 cells. (**a**–**d**) PC12-P1F1 cells were treated with 40 ng/mL bone morphogenetic protein 4 (BMP4) as a control for 7 days in the presence or absence of U0126. Representative phase-contrast images of PC12-P1F1 cells on day 7 after treatment with (**a**) 0 μM, (**b**) 0.31 μM, (**c**) 0.63 μM, and (**d**) 1.25 μM U0126. (**e**–**h**) PC12-P1F1 cells were exposed to TRTS plus SP600125 for 7 days in the presence or absence of U0126. Representative phase-contrast images of PC12-P1F1 cells on day 7 after treatment with (**e**) 0 μM, (**f**) 0.31 μM, (**g**) 0.63 μM, and (**h**) 1.25 μM U0126. Scale bars: 100 μm. (**i**,**j**) PC12-P1F1 cells were stimulated with 40 ng/mL BMP4 (**i**) or exposed to TRTS plus SP600125 (**j**) for 7 days, and the rate of neurite-bearing cells was determined on day 7. The data represent the means ± standard deviation of three replicates. ^††^ *p* < 0.01 vs. control (**i**) or TRTS alone (**j**); ** *p* < 0.01 vs. BMP4 alone (**i**) or vs. TRTS plus SP600125 only (**j**); n.s., not significant vs. TRTS alone (**j**).

Generally, the ERK1/2 signaling pathway is pivotal in regulating neurite outgrowth of PC12 parental cells [16], and the present study has already shown that MEK1/2 inhibitor U0126 also substantially inhibits TRTS-mediated neuritogenesis, even in PC12-P1F1 cells (Figure 2). Therefore, we decided to additionally examine whether the inhibition of ERK1/2 by U0126 also affects SP600125-promoted neuritogenesis induced by TRTS in PC12-P1F1 cells. For this experiment, we further investigated the dose–response effect of U0126 on the SP600125-mediated promotion of TRTS-induced neuritogenesis in PC12-P1F1 cells. After 7 days of the differentiation process, we scored the percentage of neurite-bearing cells. We used BMP4 as a control for neuronal differentiation of PC12-P1F1 cells. The results revealed that U0126 inhibited the TRTS-induced neurite outgrowth in a dose-dependent manner, even in the presence of the novel neuritogenesis enhancer SP600125 (Figure 8).

**Figure 9:**
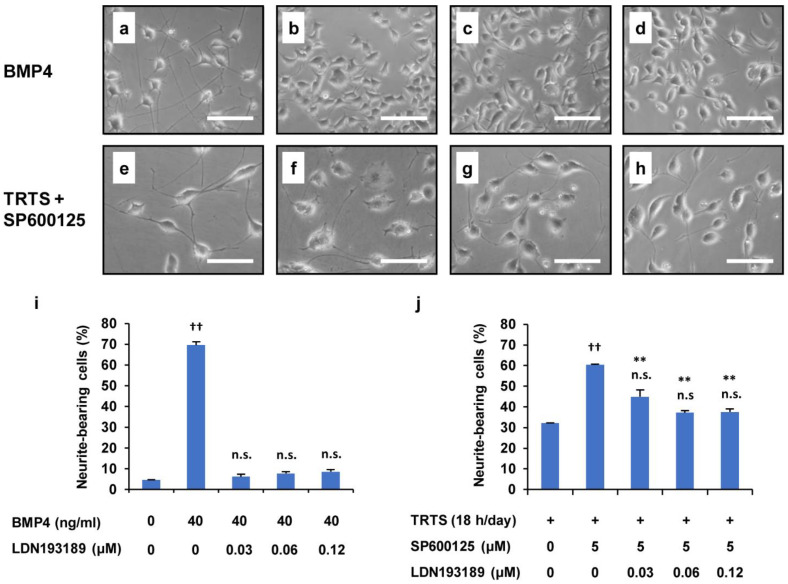
Suppression of TRTS plus SP600125-mediated neuritogenesis by LDN193189 in PC12-P1F1 cells. (**a**–**d**) PC12-P1F1 cells were treated with 40 ng/mL BMP4 as a control for 7 days in the presence or absence of LDN193189. Representative phase-contrast images of PC12-P1F1 cells on day 7 after treatment with (**a**) 0 μM, (**b**) 0.03 μM, (**c**) 0.06 μM, and (**d**) 0.12 μM LDN193189. (**e**–**h**) PC12-P1F1 cells were exposed to TRTS plus SP600125 for 7 days in the presence or absence of LDN193189. Representative phase-contrast images of PC12-P1F1 cells on day 7 after treatment with (**e**) 0 μM, (**f**) 0.03 μM, (**g**) 0.06 μM, and (**h**) 0.12 μM LDN193189. Scale bars: 100 μm. (**i**,**j**) PC12-P1F1 cells were stimulated with 40 ng/mL BMP4 (**i**) or exposed to TRTS plus SP600125 (**j**) for 7 days, and the rate of neurite-bearing cells on day 7 was determined. The data represent the means ± standard deviation of three replicates. ^††^ *p* < 0.01 vs. control (**i**) or TRTS alone (**j**); n.s., not significant vs. control (**i**) or TRTS alone (**j**); ** *p* < 0.01 vs. TRTS plus SP600125 only (**j**).

In our previous study on PC12-P1F1 cells, we undoubtedly showed that the BMP signaling inhibitor LDN193189, which selectively suppresses the kinase activity of BMP type I receptors, considerably inhibits TRTS-induced neuritogenesis in PC12-P1F1 cells, raising the possibility that TRTS-induced neuritogenesis is involved in and mediated by the BMP signaling pathway [35]. Therefore, we next investigated whether BMP signaling inhibition also affected SP600125-promoted neuritogenesis induced by TRTS in PC12-P1F1 cells. In this experiment, we calculated the percentage of neurite-bearing cells in PC12-P1F1 cells exposed to TRTS and treated with 5 μM SP600125 and the indicated concentrations of LDN193189. Cells were also treated with BMP4 alone (as a positive control) for 7 days in the presence or absence of the indicated LDN193189 concentrations. The results suggested that similar to U0126, LDN193189 also inhibits the TRTS-induced neurite outgrowth in a dose-dependent manner to some extent, even in the presence of the novel neuritogenesis promoter SP600125 (Figure 9). These results suggest that our newly developed SP600125-promoted TRTS-induced neurite elongation model still requires ERK and BMP signaling contribution.

### 2.7. Effects of TRTS in the Presence or Absence of SP600125 on Gene Expressions

In the current study, we showed that SP600125 can work as a promoter of the TRTS-mediated neuritogenesis in PC12-P1F1 cells through the atypical way in which the unknown target of SP600125 might be involved and contribute to this phenomenon. Our current data combined with our previous studies [4,35] also implied that the BMP receptor-mediated signaling might be involved in this phenomenon and TRTS-induced neuronal differentiation itself. However, most molecular mechanisms explaining the observed phenomenon remain unknown. Thus, to further elucidate these mechanisms that might contribute to further understanding the TRTS-mediated neuronal differentiation of PC12-P1F1 cells, we next attempted to clarify the effects of TRTS alone or TRTS plus SP600125 on various gene expressions. The selected genes for this study include the neuron-specific cytoskeleton marker gene β3-tubulin and the genes that encode the signaling molecule regulating the BMP receptor-mediated downstream signaling (Smad6, Smad7, p38 MAPK isoforms, and MKK3 that works as an upstream kinase of p38 MAPKs). For this experiment, the TRTS-induced differentiation of PC12-P1F1 cells was performed with or without SP600125 (5 or 10 μM, respectively) for 7 days. As in the controls, PC12-P1F1 cells were also treated with BMP4 alone (40 ng/mL) or SP600125 alone (10 μM) for 7 days. Before and after these cell treatments, the total RNA was harvested from the cells and quantitative real-time polymerase chain reaction (QRT-PCR) was performed to measure the above gene expressions in the cells.

**Figure 10:**
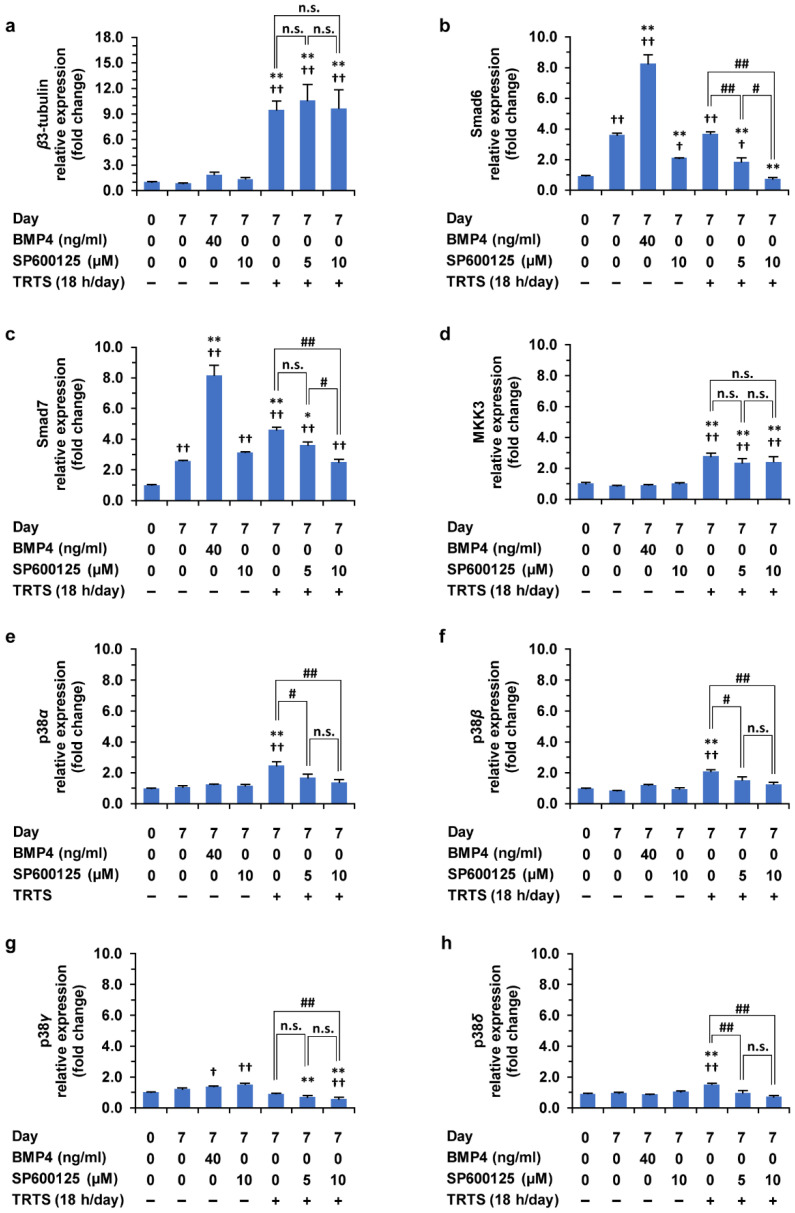
(**a**–**h**) Effects of TRTS in the presence or absence of SP600125 on gene expressions in PC12-P1F1 cells. PC12-P1F1 cells were incubated with BMP4 (40 ng/mL) or SP600125 (10.0 μM) for 7 days. In addition, PC12-P1F1 cells were also treated with TRTS for 18 h per day for 7 days in the presence of the indicated SP600125 concentrations. The target mRNA expression was normalized to that of the internal control β-actin. β3-tubulin was used as a neuronal differentiation marker of the cells. For each indicated gene, results are presented as fold changes relative to day 0 of the control: β3-tubulin (**a**), Smad6 (**b**), Smad7 (**c**), MKK3 (**d**), p38α (**e**), p38β (**f**), p38γ (**g**), p38δ (**h**). The data represent the means ± standard deviation of three replicates. ^#^ *p* < 0.05; ^##^ *p* < 0.01; ^†^ *p* < 0.05 vs. day 0 control; ^††^ *p* < 0.01 vs. day 0 control; * *p* < 0.05 vs. day 7 control; ** *p* < 0.01 vs. day 7 control; n.s., not significant.

As shown in Figure 10, β3-tubulin gene expression was found to be considerably upregulated near to tenfold after 18 h/day TRTS treatment compared to day 0 control. The β3-tubulin gene expression was also upregulated near to twofold at post-treatment with BMP4 alone for 7 days but did not statistically significantly change. These novel results are consistent with our previous observations on TRTS-mediated morphological changes, including neuritogenesis, and further strengthen the previously acquired evidence that TRTS can induce neuronal differentiation in PC12 parental cells [4]. In addition, co-treatment of SP600125 with TRTS failed to further upregulate β3-tubulin gene expression and had no significant effects on the upregulated β3-tubulin gene expression level by TRTS in PC12-P1F1 cells, increasing the possibility that the action of SP600125 in PC12-P1F1 cells might not be involved in the entire process of TRTS-induced neuronal differentiation in PC12-P1F1 cells but at least can specifically affect the process of the TRTS-mediated neurite elongation with unknown mechanisms.

**Figure 11:**
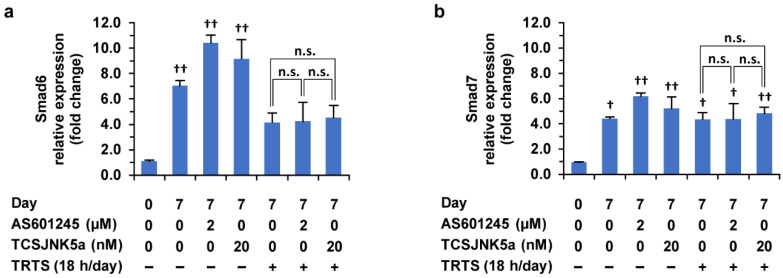
Effects of TRTS in the presence or absence of AS601245/TCSJNK5a on gene expressions in PC12-P1F1 cells. (**a**,**b**) PC12-P1F1 cells were incubated with AS601245 (2.0 μM) or TCSJNK5a (20.0 nM) for 7 days. In addition, PC12-P1F1 cells were also treated with TRTS for 18 h per day for 7 days in the presence of AS601245 (2.0 μM) or TCSJNK5a (20.0 nM). The target mRNA expression was normalized to that of the internal control β-actin. For each indicated gene, results are presented as fold changes relative to day 0 control: Smad6 (**a**), Smad7 (**b**). The data represent the means ± standard deviation of three replicates. ^†^ *p* < 0.05 vs. day 0 control; ^††^ *p* < 0.01 vs. day 0 control; n.s., not significant.

Notably, we found that Smad6 and Smad7 gene expressions were significantly upregulated (more than twofold) after 7 days of incubation in the differentiation medium compared to day 0 of the control, even without additional stimulation such as TRTS. Furthermore, Smad7 but not Smad6 gene expression was significantly upregulated near to twofold after 7 days of treatment of TRTS alone. In contrast, both Smad6 and Smad7 gene expressions were significantly upregulated more than eightfold after 7 days of treatment with BMP4 in the cells. Co-treatment of SP600125, but not of AS601245 nor TCSJNK5a (Figure 11), with TRTS significantly downregulated the gene expressions of Smad6 and Smad7. In particular, Smad6 gene expression was downregulated near the level of day 0 of control.

Regarding MKK3 gene expression, the gene expression was found to be significantly and specifically upregulated after 7 days of treatment with TRTS in the cells. However, treatment with BMP4 alone or SP600125 alone for 7 days failed to significantly upregulate the MKK3 gene expression in the cells. In addition, as in β3-tubulin gene expression, the MKK3 gene expression level was not affected by the co-treatment of SP600125 with TRTS. These results increase the possibility that the observed significant upregulation of MKK3 gene expressions after TRTS treatment might specifically contribute to the TRTS-mediated neurite genesis by activating p38 MAPK signaling in an SP600125-independent manner in PC12-P1F1 cells.

Lastly, among p38 MAPK isoforms (p38α, p38β, p38γ, and p38δ), we could detect all gene expressions for p38 MAPK isoforms in PC12-P1F1 cells and found that TRTS treatment for 7 days selectively and significantly increased gene expressions of p38α, p38β, and p38δ, but not that of p38γ. However, no significant upregulations of gene expressions for three isoforms of p38 MAPKs were observed after 7 days of treatment with BMP4 alone or SP600125 alone in the cells, as in MKK3 gene expression. Regarding the three isoforms of p38MAPK, co-treatment of SP600125 with TRTS for 7 days inhibited the upregulating effects of TRTS, and the gene expression was back near to that of day 7 in the control in the cells. In contrast, the gene expression of p38γ was differently regulated at 7 days of treatment with BMP4 or SP600125 in the cells: p38γ gene expression level was significantly upregulated at 7 days after incubation with BMP4 alone or SP600125 alone in the cells.

## 3. Discussion

This study revealed novel, important results that are useful for developing methods of regulating the efficiency of TRTS-induced neuritogenesis using PC12-P1F1 (a PC12 subclone with high sensitivity to TRTS). We found that the selective JNK inhibitor SP600125, as in BMP4, promoted TRTS-induced neurite elongation and thus enhanced TRTS-induced increase in the rate of neurite-bearing cells. In contrast, the other selective JNK inhibitors (AS601245, TCSJNK5a, and TCSJNK6o) did not enhance TRTS-induced neuritogenesis. Moreover, we also discovered that PC12-P1F1 cells pretreated with SP600125-NC (a negative control of SP600125) enhanced TRTS-induced neuritogenesis to a similar degree as that of SP600125 itself. Additionally, we found that SP600125-enhanced TRTS-induced neuritogenesis in the PC12-P1F1 cells was still substantially attenuated by treatment with the MEK1/2 inhibitor U0126 or the BMP receptor inhibitor LDN193189. In this context, the current study also showed the ability of both inhibitors to suppress the neurite outgrowth induced by TRTS alone, respectively. With this viewpoint, gene expression analysis using QRT-PCR that was further performed to clarify the relationship between the SP600125-mediated unknown signaling pathway(s) during TRTS-induced neuritogenesis and the BMP signaling pathways first revealed that TRTS can increase not only a neuronal differentiation marker and β3-Tubulin gene expression but also a part of important BMP-signaling mediators, MKK3 and Smad7 in the cells. Moreover, Smad6 and Smad7 gene expressions were found to be substantially attenuated through co-treatment with SP600125, but not the other JNK inhibitors (AS601245 and TCSJNK5a), after TRTS treatment for 7 days. Owing to the fact that BMP signaling is known to be involved in BMP-mediated neuritogenesis in PC12 cells [19,29] and I-Smads are well-known negative regulators of BMP signaling itself [27,28,31], these results suggest that SP600125 might specifically work as an enhancer of TRTS-induced neuritogenesis in a JNK signaling pathway-independent manner during the process of TRTS-induced neuronal differentiation, at least in part by attenuating the I-Smad-mediated negative feedback loop of BMP signaling in the cells.

Regarding the effects of the employed chemical inhibitors on TRTS-induced neuronal differentiation, we had previously shown that p38 MAPK and ERK1/2 signaling pathways were required for TRTS-mediated neurite outgrowth of PC12 parental cells [4,35], but the role of JNK remained unclear. In the present study, we first investigated the effects of the inhibitors of every MAPK family member, including commercially available JNK selective inhibitors (SP600125, AS601245, TCSJNK5a, and TCSJNK6o), on TRTS-induced neuritogenesis in PC12-P1F1 cells. The results revealed that TRTS-induced neuritogenesis in PC12-P1F1 was significantly decreased in the presence of p38 MAPK inhibitor SB2003580, U0126, and MEK5 inhibitor BIX02189. These observations are consistent with previous reports that ERK1/2 and p38 MAPK probably play roles in TRTS differentiation in PC12 parental cells [4]. Further, in our present study, we described first that most JNK inhibitors (AS601245, TCSJNK5a, and TCSJNK6o) did not obviously affect TRTS-induced neuritogenesis, suggesting that the JNK signaling pathway might not play an essential role in TRTS induced-neuronal differentiation in PC12-P1F1 cells. However, in our present study, we additionally observed the unexpected promotion of TRTS-mediated neuritogenesis by the remaining JNK selective inhibitor SP600125. The underlying molecular mechanism of this phenomenon is partially determined in this study as described above using gene expression analysis with QRT-PCR, but its total elucidation might reveal novel methods for further improving the efficacy of TRTS for future applications, particularly in the area of nerve regeneration medicine, because, to our knowledge, only SP600125 and its analog (SP600125-NC) enhance the efficiency of TRTS-mediated neuritogenesis as chemical inhibitors.

Regarding the future molecular analysis of the effects of SP600125 and the other JNK inhibitors on TRTS-mediated neuronal differentiation, it should be noted that the present study successfully demonstrated that the enhancement of TRTS-induced neuritogenesis and the inhibition of I-Smad gene expression by SP600125 occurred in a dose-dependent manner. However, our investigation of various treatment times of PC12-P1F1 cells with SP600125 failed to identify an optimal time that significantly promoted the effects of SP600125 on TRTS-induced neuritogenesis, suggesting that continuous treatment of the cells with SP600125 subsequently induces the attenuation of I-Smad gene expressions that might be important to further clarify the unknown mechanism of the enhancing effects of SP600125.

Regarding the concrete molecular action of SP600125 in the cells, it should be also noted that SP600125 was originally characterized as a selective ATP-competitive inhibitor of JNKs [36]. SP600125 has a highly planar structure and a nitrogen-containing ring system for interaction with key residues in the kinase active site of JNKs. The function of JNK inhibition is related to its pyrazole moiety, which forms a critical hydrogen-bonding interaction at the ATP-binding site of JNK. In SP600125-NC, the critical free NH is replaced by an N-alkyl substituent; thus, theoretically, SP600125-NC does not possess the ability to interact with and inhibit JNK at the same concentration levels as SP600125. In the present study, our results clearly showed that 5 μM SP600125-NC also conferred a similar enhancing effect on TRTS-mediated neuritogenesis in PC12-P1F1 cells. Thus, our results evidently suggest that the suppression of the JNK signaling pathway by SP600125 might not be the trigger that induces the observed promoting effect of SP600125 in PC12-P1F1 cells. Moreover, our observations in the present study also suggest that the true key structure within SP600125 that is essential for the SP600125-mediated promotion of neurite genesis is not the pyrazole moiety (which is included in SP600125 and inhibits JNK signaling). Rather, it may be a structure common to both SP600125 and SP600125-NC, and this should be elucidated in a future study.

In this context, regarding the atypical action of SP600125, Vaishnav et al. have demonstrated that in addition to inhibiting the JNK signaling pathway, SP600125 rapidly activated cAMP response element-binding protein (CREB) by activating the p38 MAPK-mediated signaling pathway in the mouse β-cell line MIN6 [37]. This reported phenomenon might help us to determine the true target(s) of the observed promoting effect of SP600125 because, as described above, a study on BMP2 and PC12 parental cells indicated that BMP-mediated activation of the p38 MAPK signaling pathway alone significantly induced neuronal differentiation of the cells [19,29]. Additionally, Bonni et al. reported the potential of CREB to promote the survival and differentiation of neurons because it exhibited a specificity to neurotrophin signals [38]. Thus, regarding the results of the present study that 10 μM SP600125 alone induced neuritogenesis to some extent, the combination of the above reports on intracellular signaling mechanisms raises the possibility that the relatively higher concentration (10 μM) of SP600125 that we employed might induce the neuritogenesis of PC12-P1F1 cells through CREB activation by SP600125-mediated p38 MAPK phosphorylation. Therefore, as one of the working hypotheses for a future study, it may be worthwhile to assess whether the SP600125-mediated promoting effects on TRTS-induced neuritogenesis may partially be caused by the SP600125-mediated elevation of the p38 MAPK activity followed by CREB activation. In this context, we first showed that, among the four isoforms of p38 MAPK, only p38γ gene expression was significantly upregulated by the treatment of SP600125 alone for 7 days. Therefore, additional study on the role of p38γ in the effects of SP600125 on the TRTS-induced neuritogenesis might contribute to the understanding of the action of SP600125 in PC12-P1F1 cells.

On the other hand, regarding the relationship between the ERK1/2 signaling pathway and BMP or TRTS-induced neuritogenesis, the present study is the first to report that U0126, an inhibitor of ERK1/2, inhibits neurite outgrowth induced by BMP4 in PC12-P1F1 cells as if it were an inhibitor of BMP signaling itself, supporting the idea that ERK1/2 signaling may also participate in the process of BMP-mediated neuritogenesis. We also first observed the U0126-mediated partial attenuation of neuritogenesis induced by TRTS plus SP600125, which proposes whether the ERK1/2 signaling pathway is independent of the SP600125-mediated promotion system of TRTS-induced neuritogenesis in the cells or not and further investigation using gene expression analysis, such as performed in the current study for analyzing the BMP receptor-mediated signaling pathway, might contribute to elucidate the issue. 

Regarding the involvement of the BMP signaling pathway in TRTS-induced neuritogenesis, our previous study has already reported that the use of LDN193189 to inhibit the BMP signaling pathway at the level of the membrane receptors could significantly block TRTS-mediated neuritogenesis in PC12-P1F1 cells [35]. Furthermore, in the present study, we first showed that the treatment of PC12-P1F1 cells with TRTS alone for 7 days significantly increase the gene expressions of MKK3 and Smad7, important mediators of BMP-receptor mediated p38 MAPK signaling. Moreover, we demonstrated that co-treatment of BMP4 with TRTS for 7 days could efficiently enhance TRTS-mediated neuritogenesis in the cells. Therefore, the involvement of the BMP receptor-meditated p38 MAPK signaling pathway in TRTS-induced neuritogenesis in a complex manner cannot be denied. However, in this context, we observed pretreating the cells with LDN193189 thoroughly blocked the neurite outgrowth induced by BMP4 (used as a control), even in a very limited concentration in the present study. Meanwhile, the same concentrations of LDN193189 actually decreased the SP600125-mediated promotion of TRTS-induced neuritogenesis to some extent. Interestingly, the differentiation rate that reflects the percentage of neurite-bearing cells in the presence of LDN193189 decreased only near that for the cells treated with TRTS alone. Thus, these results also imply that the cells exposed to TRTS alone or TRTS plus SP600125 treatment may activate multiple signaling pathways at the same time in addition to BMP signaling.

## 4. Materials and Methods

### 4.1. Cells and Reagents

Rat pheochromocytoma PC12 cells (PC12 parental cells) were supplied by RIKEN BioResource Research Center (Tsukuba, Japan). PC12-P1F1 and P1D10 subclones that we had previously isolated [35] were stored in liquid nitrogen (Saisan Corp., Ltd., Saitama, Japan). Penicillin/streptomycin solution was purchased from Sigma-Aldrich (St. Louis, MO, USA). Glutamic acid, NaCl, glycine, and sucrose were from FUJIFILM Wako Pure Chemical Corp. (Tokyo, Japan). Tween 80 was obtained from MP Biomedicals (Solon, OH, USA). Recombinant human BMP4 (PeproTech, Rocky Hill, NJ, USA) and recombinant human ꞵ-NGF (PeproTech) were dissolved in LF6 buffer solution (5 mM glutamic acid, 5 mM NaCl, 2.5% glycine, 0.5% sucrose, and 0.01% Tween 80; pH 4.5). LDN193189 (a selective inhibitor of BMP type I receptors), BIX02189 (a selective inhibitor of MEK5), selective JNK inhibitors (AS601245, TCSJNK5a, TCSJNK6o, and SP600125), and SP600125 negative control (SP600125-NC), a negative control chemical for SP600125, were obtained from Cayman Chemical (Ann Arbor, MI, USA). TCSJNK6o and AS601245 are inhibitors of JNK1, JNK2, and JNK3, and TCSJNK5a is a selective inhibitor of JNK2 and JNK3 [39]. The MEK1/2-specific inhibitor U0126 was obtained from Calbiochem (San Diego, CA, USA). The p38 MAPK-specific inhibitor SB203580 was obtained from Enzo Life Sciences (Farmingdale, NY, USA). The above chemical inhibitors were dissolved in dimethyl sulfoxide (FUJIFILM Wako Pure Chemical Corp.).

### 4.2. Cell Culture and the Induction of Neurite Outgrowth

PC12 parental cells and the subclones PC12-P1F1 and PC12-P1D10 were cultured in Dulbecco’s modified Eagle’s medium (DMEM; FUJIFILM Wako Pure Chemical Corp.) supplemented with 10% FetalClone III artificial serum (Cytiva, UT, USA) and penicillin/streptomycin and incubated at 37 °C in an atmosphere of 5% CO_2_. For the neuritogenesis assay, we seeded cells at a density of 1.5 × 10^4^ cells/well in collagen type IV-coated 24-well plates (Corning, Corning, NY, USA) with 0.5 mL medium/well. After incubation for 24 h, the cells were serum-starved in DMEM supplemented with 1% FetalClone III and penicillin/streptomycin and placed on heating plates (Thermoplate II or III; Tokai Hit, Fujinomiya, Japan) to maintain the surface temperature during TRTS. In this context, ThermoPlate III, primarily used in the current study, is the successor to Thermoplate II, previously discontinued by the manufacturer. For TRTS, the culture medium was heated to approximately 39 °C, which has been determined as the optimal temperature for neuronal differentiation [4], for a total of 18 h per day. In our previous studies, 3 h of heat stimulation was repeated six times (total = 18 h/day) for 7 days [4], and we further developed a modified TRTS program of 9 h twice a day for 7 days [35]. In this study, we modified the TRTS program to make it simpler to use by exposing the cells to sustained heat for 18 h per day for 7 days. After heating for 18 h, we programmed a break of 6 h using a PT70DW digital timer (REVEX, Kawaguchi, Japan), which digitally controls the on/off of power period of the device to which it is connected. As positive controls for neuritogenesis, we treated cells with 50 ng/mL β-NGF or 40 ng/mL BMP4 [35] and quantified neurite outgrowth as previously described [4,28,35]. Briefly, neuritogenesis was assessed using a Leica DM IL LED phase-contrast microscope (Leica Microsystems, Wetzlar, Germany). Three or four random images per well were taken using an MC120 HD digital camera (Leica Microsystems) connected to Leica Application Suite v4.5 image capture software (Leica Microsystems). Cells with projections 1.5 times longer than the maximum cell body length were defined as positive for neuritogenesis. For a cell bearing multiple projections or a projection with branches, the longest neurite length per cell was selected for evaluation. At least 70 cells were evaluated per well. Each data point corresponded to the counts acquired from three or four independent wells.

### 4.3. Thermal Evaluation of the Medium

We used a temperature sensor (miniature thermocouple) associated with the heating plate (Thermoplate III) to measure the temperature of the medium in the presence or absence of TRTS. The apical end of the sensor was placed into a cell-free growth medium (0.5 mL/well) in 24-well culture plates. For temperature equilibration, the 24-well culture plate was placed on a heating plate at 37 °C in an E-50 CO_2_ incubator (As One Corp., Osaka, Japan) 24 h before TRTS treatment. Subsequently, the culture medium on the heating plate was exposed to TRTS for 18 h per day for 7 days, and the thermal change in the culture medium in the plate was monitored every 60 s. The temperature in the medium during TRTS was recorded by a TC-08 thermocouple data logger and PicoLog 6 data logging software (both from Pico Technology, Cambridge, UK).

### 4.4. Live Cell Imaging with Fluorescein Diacetate Staining

For live cell imaging, fluorescein diacetate staining assay was performed as described previously [40]. The cells were incubated at 37 °C in an atmosphere of 5% CO_2_ and examined on days 0 and 7 during the above-described neuritogenesis assay. Fluorescein diacetate (FDA; Dojindo Laboratories, Kumamoto, Japan) was added to experimental wells at the final concentration of 5 μM in a culture medium on the days of testing. Then, the cells were incubated at 37 °C in an atmosphere of 5% CO_2_ again under a light-shading condition. After the incubation, the culture medium including FDA was removed from each experimental well, and then fresh culture medium was added again to each experimental well. Live cells stained with FDA were visualized and captured as digital data using fluorescence microscopy (FLoid Cell imaging station; Thermo Fisher Scientific, Waltham, MA, USA).

### 4.5. Neurite Length Measurement of Live Cells

The lengths of individual neurites for each live cell were measured using a tracing method and Microsoft Office PowerPoint (Microsoft, Seattle, WA, USA). In detail, for this measurement, the length of the scale bar specifying the scale (in microns) included in the FDA-stained digital fluorescent images of the cells was at first digitally traced and measured in millimeters using drawing tools of the employed software, and the corresponding conversion factor was determined. Then, the respective neurite lengths included in neuritogenesis-positive cells were measured in millimeters. The acquired neurite length in millimeters was then converted back to microns using the calculated conversion factor. Neuritogenesis-negative cells were excluded from this measurement. Neurite lengths were measured by digitally tracing each neurite from the neurite base along the usually curved neurites to the neurite tips. For the tracing, the length of the scale bar or each neurite, one or multiple digital straight line(s) were used and manually merged on the aim of the scale bar or neurite using the PowerPoint, and the total length of the line(s) was calculated using Microsoft Office Excel (Microsoft). In the case of branched neurites, the length of the longest branch was selected and measured to determine the longest length for each neurite.

### 4.6. Quantitative Real-Time Polymerase Chain Reaction

RNA was isolated using an RNeasy Mini kit (Qiagen, Strasse, Germany) and QIA shredder columns (Qiagen), and the first-strand cDNA was synthesized with 50 ng of RNA using the Super Script III reverse transcriptase (Thermo Fisher Scientific) and Random Primers (Promega, Madison, WI, USA). QRT-PCR was performed with the Power SYBR Green Master Mix (Thermo Fisher Scientific) as previously described [41]. The primers were purchased from Takara Bio (Shiga, Japan) and are shown in Table 1. PCR signals were detected with CFX Connect (Bio-Rad, Berkeley, CA, USA) based on the manufacturer’s instructions. All expression data were normalized to the levels of the internal control β-actin.

### 4.7. Statistical Analysis

The data were presented as the mean ± standard deviation. Significant differences between groups were identified by one-way analysis of variance followed by the Holm test or two-way analysis of variance followed by the Bonferroni test as appropriate. Statistical analysis was performed with the statistical package JSTAT v22.0J for Windows (Sato, Japan). *p*-values < 0.05 were considered statistically significant.

## 5. Conclusions

In conclusion, this is the first report of the identification and characterization of the novel promoting effect of SP600125, an extremely well-known chemical compound that selectively inhibits JNK, on TRTS-mediated neuritogenesis in PC12-P1F1 cells. Notably, regarding the mechanism of this phenomenon, we used SP600125-NC to unequivocally demonstrate that the JNK signaling pathway itself is not essential for the promotion effect of SP600125 on TRTS-induced neuritogenesis in PC12-P1F1 cells. The above new knowledge implies the possible existence of a novel unknown target biomolecule in PC12-P1F1 cells that has a selective affinity to a structure common to both SP600125 and SP600125-NC. In addition, further investigation to reveal the detailed underlying molecular mechanisms showed (i) significant upregulations not only in the β3-tubulin gene, but also that of the MKK3 gene, three isoforms of p38 MAPK gene (p38α, p38β, and p38δ, but not p38γ), and Smad7 gene after 7 days of treatment with TRTS alone. (ii) Moreover, we also clearly showed the partial downregulation of Smad6 and Smad7 gene expression levels in addition to the partial downregulation of the gene expression level for each p38 MAPK isoform after co-treatment of SP600125 with TRTS, which, for example, increases a possibility that the strengthening of p38 MAPK signaling via the observed upregulation of MKK3 gene after the TRTS treatment and the induced attenuation of I-Smad gene expressions after the co-treatment of SP600125 with TRTS that might promote inhibition of negative feedback regulation of BMP receptor-mediated signaling after the co-treatment of SP600125 with TRTS. Taken together, the novel findings in the present study will promote the expansion of our understanding of the effects of SP600125 in the course of TRTS-mediated neuritogenesis in PC12-P1F1 cells and are highly valuable for the development of future regenerative neuromedicine involving TRTS.

## Figures and Tables

**Table 1:** Primer sequences for quantitative real-time polymerase chain reaction (QRT-PCR).

Genbank Accession Number	Gene	Forward Primer (5′-3′)	Reverse Primer (5′-3′)
NM_139254.2	β3-tubulin	TCCACCTTCATCGGCAACA	CGGTGAACTCCATCTCATCCA
NM_001109002.3	Smad6	CACTGCTCCGGGTGAATTCTC	AGTATGCCACGCTGCACCA
NM_030858.2	Smad7	AGCAAGAGTCAGCACTGCCAAG	TGACAACTGAAATGCTGATCCAAAG
NM_001100674.1	MKK3	GTCTGGAGCCTTGGCATCAC	CCTGCTTCAGCTGCTGGAAC
NM_031020.3	p38α	ATGCAGTCCAGCTCCACGTC	TCCTAACACAGCATGGCCACA
NM_001109532.2	p38β	GGCAAAGATATCCTCGGAGCA	TGGTCACTGTCTAGCACCAGCA
NM_021746.2	p38γ	TGGCTGTGAACGAGGACTGTG	GATGACCTCTGGTGCCCGATA
NM_019231.3	p38δ	GAAGGTCCAGTATTTGGTGTACCAG	CTTCATTCACGGCCAGGTTG
NM_031144.3	β-actin	GGAGATTACTGCCCTGGCTCCTA	GACTCATCGTACTCCTGCTTGCTG

## Data Availability

The data presented in this study are available in the article.

## References

[1] Teng K.K., Georgieff I.S., Nunez J., Shelanski M.L., Greene L.A. (1993). Characterization of a PC12 cell sub-clone (PC12-C41) with enhanced neurite outgrowth with capacity: Implications for a modulatory role of high molecular weight tau in neuritogenesis. J. Cell Sci..

[2] Gokoffski K.K., Peng M., Alas B., Lam P. (2020). Neuro-protection and neuro-regeneration of the optic nerve: Recent advances and future directions. Curr. Opin. Neurol..

[3] Higgins S., Lee J.S., Ha L., Lim J.Y. (2013). Inducing neurite outgrowth by mechanical cell stretch. Biores. Open Access.

[4] Kudo T., Kanetaka H., Mochizuki K., Tominami K. (2015). Induction of neurite outgrowth in PC12 cells treated with temperature-controlled repeated thermal stimulation. PLoS ONE.

[5] Wiatrak B., Kubis-Kubiak A., Piwowar A., Barg E. (2020). PC12 cell line: Cell types, coating of culture vessels, differentiation and other culture conditions. Cells.

[6] Radio N.M., Mundy W.R. (2008). Developmental neurotoxicity testing in vitro: Models for assessing chemical effects on neurite outgrowth. Neurotoxicology.

[7] Greene L.A., Tischler A.S. (1976). Establishment of a noradrenergic clonal line of rat adrenal pheochromocytoma cells which respond to nerve growth factor. Proc. Natl. Acad. Sci. USA.

[8] Gill J.S., Schenone A.E., Podratz J.L., Windebank A.J. (1998). Autocrine regulation of neurite outgrowth from PC12 cells by nerve growth factor. Mol. Brain Res..

[9] Mielke K., Herdegen T. (2000). JNK and p38 stresskinases--degenerative effectors of signal-transduction-cascades in the nervous system. Prog. Neurobiol..

[10] Ambrosino C., Mace G., Galban S., Fritsch C., Vintersten K., Black E., Gorospe M., Nebreda A.R. (2003). Negative feedback regulation of MKK6 mRNA stability by p38alpha mitogen-activated protein kinase. Mol. Cell. Biol..

[11] Ana C., Angel R.N. (2010). Mechanisms and functions of p38 MAPK signalling. Biochem. J..

[12] Raingeaud J., Whitmarsh A.J., Barrett T., Dérijard B., Davis R.J. (1996). MKK3- and MKK6-regulated gene expression is mediated by the p38 mitogen-activated protein kinase signal transduction pathway. Mol. Cell. Biol..

[13] Burton J.C., Grimsey N.J. (2019). Ubiquitination as a Key Regulator of Endosomal Signaling by GPCRs. Front. Cell Dev. Biol..

[14] Johnson G.L., Lapadat R. (2002). Mitogen-activated protein kinase pathways mediated by ERK, JNK, and p38 protein kinases. Science.

[15] Cargnello M., Roux P.P. (2011). Activation and function of the MAPKs and their substrates, the MAPK-activated protein kinases. Microbiol. Mol. Biol. Rev..

[16] Vaudry D., Stork P.J.S., Lazarovici P., Eiden L.E. (2002). Signaling pathways for PC12 cell differentiation: Making the right connections. Science.

[17] Obara Y., Yamauchi A., Takehara S., Nemoto W., Takahashi M., Stork P.J.S., Nakahata N. (2009). ERK5 activity is required for nerve growth factor-induced neurite outgrowth and stabilization of tyrosine hydroxylase in PC12 cells. J. Biol. Chem..

[18] Kashino Y., Obara Y., Okamoto Y., Saneyoshi T., Hayashi Y., Ishii K. (2018). ERK5 phosphorylates Kv4.2 and inhibits inactivation of the A-type current in PC12 cells. Int. J. Mol. Sci..

[19] Iwasaki S., Iguchi M., Watanabe K., Hoshino R., Tsujimoto M., Kohno M. (1999). Specific Activation of the p38 mitogen-activated protein kinase signaling pathway and induction of neurite outgrowth in PC12 cells by bone morphogenetic protein-2. J. Biol. Chem..

[20] Liu J., Lin A. (2005). Role of JNK activation in apoptosis: A double-edged sword. Cell Res..

[21] Xiao J., Zhou Q., Liu Y. (2002). Variant PC12 cell line that spontaneously differentiates and extends neuritic processes. J. Neurosci. Res..

[22] Xiao J., Pradhan A., Liu Y. (2006). Functional role of JNK in neuritogenesis of PC12-N1 cells. Neurosci. Lett..

[23] Ebendal T., Bengtsson H., Söderström S. (1998). Bone morphogenetic proteins and their receptors: Potential functions in the brain. J. Neurosci. Res..

[24] Iwasaki S., Hattori A., Sato M., Tsujimoto M., Kohno M. (1996). Characterization of the bone morphogenetic protein-2 as a neurotrophic factor. Induction of neuronal differentiation of PC12 cells in the absence of mitogen-activated protein kinase activation. J. Biol. Chem..

[25] Althini S., Usoskin D., Kylberg A., Kaplan P.L., Ebendal T. (2004). Blocked MAP kinase activity selectively enhances neurotrophic growth responses. Mol. Cell. Neurosci..

[26] Lönn P., Zaia K., Israelsson C., Althini S., Usoskin D., Kylberg A., Ebendal T. (2005). BMP enhances transcriptional responses to NGF during PC12 cell differentiation. Neurochem. Res..

[27] Kimura N., Matsuo R., Shibuya H., Nakashima K., Taga T. (2000). BMP2-induced apoptosis is mediated by activation of the TAK1-p38 kinase pathway that is negatively regulated by Smad6. J. Biol. Chem..

[28] Kudo T., Kanetaka H., Mizuno K., Ryu Y., Miyamoto Y., Nunome S., Zhang Y., Kano M., Shimizu Y., Hayashi H. (2011). Dor somorphin stimulates neurite outgrowth in PC12 cells via activation of a protein kinase A-dependent MEK-ERK1⁄2 signaling pathway. Genes Cells.

[29] Yanagisawa M., Nakashima K., Takeda K., Ochiai W., Takizawa T., Ueno M., Takizawa M., Shibuya H., Taga T. (2001). Inhibition of BMP2-induced, TAK1 kinase-mediated neurite outgrowth by Smad6 and Smad7. Genes Cells.

[30] Hirata Y., Takahashi M., Morishita T., Noguchi T., Matsuzawa A. (2017). Post-Translational Modifications of the TAK1-TAB Complex. Int. J. Mol. Sci..

[31] Kudo T., Kanetaka H., Watanabe A., Okumoto A., Asano M., Zhang Y., Zhao F., Kano M., Shimizu Y., Tamura S. (2010). Investigating bone morphogenetic protein (BMP) signaling in a newly established human cell line expressing BMP receptor type II. Tohoku. J. Exp. Med..

[32] Long Q., Wu B., Yang Y., Wang S., Shen Y., Bao Q., Xu F. (2021). Nerve guidance conduit promoted peripheral nerve regeneration in rats. Artif. Organs.

[33] Kano Y., Nohno T., Takahashi R., Hasegawa T., Hiragami F., Kawamura K., Motoda H., Sugiyama T. (2001). CAMP and calcium ionophore induce outgrowth of neuronal processes in PC12 mutant cells in which nerve growth factor-induced outgrowth of neuronal processes is impaired. Neurosci. Lett..

[34] Murai H., Hiragami F., Kawamura K., Motoda H., Koike Y., Inoue S., Kumagishi K., Ohtsuka A., Kano Y. (2010). Differential response of heat-shock-induced p38 MAPK and JNK activity in PC12 mutant and PC12 parental cells for differentiation and apoptosis. Acta Med. Okayama.

[35] Kudo T., Tominami K., Izumi S., Hayashi Y., Noguchi T., Matsuzawa A., Hong G., Nakai J. (2020). Characterization of PC12 cell subclones with different sensitivities to programmed thermal stimulation. Int. J. Mol. Sci..

[36] Bennett B.L., Sasaki D.T., Murray B.W., O’Leary E.C., Sakata S.T., Xu W., Leisten J.C., Motiwala A., Pierce S., Satoh Y. (2001). SP600125, an anthrapyrazolone inhibitor of jun N-terminal kinase. Proc. Natl. Acad. Sci. USA.

[37] Vaishnav D., Jambal P., Reusch J.E.B., Pugazhenthi S. (2003). SP600125, an inhibitor of c-Jun N-terminal kinase, activates CREB by a p38 MAPK-mediated pathway. Biochem. Biophys. Res. Commun..

[38] Bonni A., Ginty D.D., Dudek H., Greenberg M.E. (1995). Serine 133-phosphorylated CREB induces transcription via a cooperative mechanism that may confer specificity to neurotrophin signals. Mol. Cell. Neurosci..

[39] Angell R.M., Atkinson F.L., Brown M.J., Chuang T.T., Christopher J.A., Cichy-Knight M., Dunn A.K., Hightower K.E., Malkakorpi S., Musgrave J.R. (2007). N-(3-Cyano-4,5,6,7-Tetrahydro-1-Benzothien-2-Yl)amides as potent, selective, inhibitors of JNK2 and JNK3. Bioorganic Med. Chem. Lett..

[40] Nunome S., Kanetaka H., Kudo T., Endoh K., Hosoda H., Igarashi K. (2015). In vitro evaluation of biocompatibility of Ti-Mo-Sn-Zr superelastic alloy. J. Biomater. Appl..

[41] Hayashi Y., Otsuka K., Ebina M., Igarashi K., Takehara A., Matsumoto M., Kanai A., Igarashi K., Soga T., Matsui Y. (2017). Distinct requirements for energy metabolism in mouse primordial germ cells and their reprogramming to embryonic germ cells. Proc. Natl. Acad. Sci. USA.

